# Surgical site infections following abdominal surgery: incidence, risk factors and outcomes

**DOI:** 10.4314/ahs.v24i4.12

**Published:** 2024-12

**Authors:** Olufemi O Ojewuyi, Abiodun R Ojewuyi, Adeniyi O Fasanu, Opeyemi Q Asafa, Oluwaseun A Taiwo, Emmanuel O Folami

**Affiliations:** 1 Department of Surgery, UNIOSUN Teaching Hospital and College of Health Sciences, Osun State University, Osogbo; 2 Department of Medical Microbiology and Parasitology, UNIOSUN Teaching Hospital and College of Health Sciences, Osun State University, Osogbo; 3 Department of Obstetrics and Gynaecology, UNIOSUN Teaching Hospital and College of Health Sciences, Osun State University, Osogbo; 4 Department of Anaesthesia, UNIOSUN Teaching Hospital and College of Health Sciences, Osun State University, Osogbo

**Keywords:** surgical site infections, Abdominal Surgery

## Abstract

**Background:**

Abdominal surgeries especially open surgeries are associated with high risk of surgical site infections. This invariably leads to increased morbidity, prolonged hospital stay with its attendant financial burden and mortality.

**Objectives:**

To describe the incidence, risk factors and outcome in patient with surgical site infection following open abdominal surgery.

**Methods:**

This was a prospective study involving consecutive 100 patients across surgical sub-specialties and gynaecology over 6 month period. Variables analyzed included; biodata, type of surgery, American Society of Anaesthesiologists (ASA) grade, hair removal at wound site, specialty, degree of intraoperative contamination, presence of surgical site infection (SSI) and mortality.

**Results:**

Majority of the patients (64%) were women and the age range was 11 to 73 years, mean age was 36.41 ± 10.9 years. About 60% of the cases were emergency procedures, indication for surgery were benign in 89 (89%) patients, malignant in 7% and trauma in 4%. Majority (55%) had clean-contaminated wounds while 45% had contaminated and dirty wounds, 54% were solely general surgery patients, 66% of the patients had hair removal before theatre arrival. SSI was recorded in 20% of the patients and mortality rate was 7% at 30 days follow up.

The risk of SSI was higher in emergency procedures, (p =0.041) with an odd ratio of 2. SSI risk increases with the ASA grade, general surgical procedures, hair removal at wound site before theatre arrival and also in contaminated and dirty wounds. Emergency procedures and SSI were found to increase the odds of mortality.

**Conclusion:**

Emergency procedures, general surgery (bowel surgeries), and significant degrees of intraoperative contamination are associated with higher risk of SSI, which is an independent predictor of mortality. More efforts should be put in place to prevent SSI in these categories of patients.

## Introduction

Surgical Site Infections (SSI) is defined by the Centre for Disease Control and Prevention (CDC) as any infection along the surgical tract which occurs within 30 days of an operative procedure or within a year if an implant is left-in-situ and the infection is thought to be related to surgery^1^. It is categorized into a superficial incisional SSI, deep incisional SSI and organ space SSI. In comparison to other types of surgical interventions, the rates of SSI have been found to be higher in abdominal surgery, some prospective studies have reported an incidence of between 15%-25% depending on the level of contamination^2^-^4^. Surgical site infection though preventable, is associated with high morbidity and mortality^5^. The Centre for Disease Control and Prevention recommended measures aimed at decreasing the risk of SSI. These include; preoperatively; optimal blood sugar control, cessation of smoking, lessening preoperative stay, intraoperatively; strict asepsis, moderate use of antibiotics, effective haemostasis, obliterating dead spaces, proper tissue handling, correct placement of drain, postoperatively; aseptic approach to wound care. Some have also considered laparoscopic approach to be associated with lesser risk of SSI^1^. However, the incidence of SSI is still high^6^. While SSI is regarded as a global issue irrespective of the access of surgery, it is one of the most common healthcare-associated infection in low- and middle-income countries (LMIC)^1^,^7^. It has a devastating impact on the patients and family, unduly prolongs their hospital stay in addition to the high costs^8^,^9^. In Nigeria, insurance services are limited and majority of our patients accessing healthcare facilities pay out of pocket. Thus, SSI increases the financial burden on the patient and relatives. The goal of this study was to describe the factors associated with SSI, the incidence and outcome in patients who had open abdominal surgery at our hospital.

## Methods

We conducted a prospective study at UNIOSUN Teaching Hospital, Osogbo, Osun State Nigeria. The target populations were patients above the age of 5 years who underwent open abdominal surgery. Patients were recruited consecutively for a period of 6 months, from October 2021 to March 2022. Patients were identified immediately preoperatively and recruited into the study after obtaining informed consent. Recruitments were done across general surgery, urology, paediatric surgery and gynaecology. Patients below the age of 5 years and those whose surgery were classified as clean wound were excluded. Institutional ethical approval was obtained. A study proforma was used to collect necessary information. Data collected include; demographic characteristics, preoperative risk factors - diabetes status, smoking status, ASA classification, indication for surgery. Intraoperative: timing of perioperative antibiotics, timing of hair removal where applicable, type of surgery, specialty, incision approach, degree of intraoperative contamination, presence of SSI and outcome. Patients were followed up postoperatively for 30 days (while on the ward, during outpatient clinic visits and via telephone interview) to look out for surgical site infection. Diagnostic criteria used to determine presence of wound infection were based on the CDC criteria; purulent exudation, redness, heat, local swelling and tenderness, fever, abscess, spontaneous wound dehiscence or deliberately opened incision by surgeon with or without culture. Presence of at least one of the above is enough to make a diagnosis of SSI (CDC). Age was presented with mean and standard deviation while categorical variables were presented with frequency and percentages. Chi square and Fischer exact test were used to determine association between categorical variables while independent student t-test was used for comparison between means. Multivariate logistic regression was used to assess independent predictor of surgical site infection and mortality outcome. Parameters with pvalue <0.2 in binary regression were inputted into multivariate logistic regression. P value <0.05 was assumed significant at 95% confidence interval.

## Results

In total, 100 patients were enrolled in the study, 64 females (64%) and 36 males (36%) with an overall mean age of 36.4 years. They were all non-smokers, only 3 (3%), all males were diabetic. They all had intravenous antibiotics 1 hour prior to the incision time. Elective procedures were 40 (40%), while 60 patients (60%) had emergency surgery. Indications were majorly benign, 89 (89%), malignant in 7 (7%) and traumatic in 4 (4%) patients. Majority, 85 (85%) had ASA 1 and 11, operative approach was open midline in 51 (51%) and open non-midline in 49 (49%). Based on the degree of intraoperative contamination, 55 (55%) had clean contaminated, 33 (33%) and 12 (12%) had contaminated and dirty wound respectively. Majority (54%) were solely general surgery (bowel surgery) patients, 66% of patients had hair removal at wound site before theatre arrival. Table 1.

**Table 1:** Clinical characteristic of participants

	Male (n=36)	Female (n=64)	Overall	p-value
**Age in years (Mean±SD)**	37.28±10.3	35.92±11.7	36.41±10.9	0.669
**Diabetes Mellitus status**				
Present	3(8.3)	0(0.0)	3(3.0)	0.019*
Absent	33(91.7)	64(100.0)	97(97.0)	
**Type of surgery**				0.307
Elective	12(33.3)	28(43.8)	40(40.0)	
Emergency	24(66.7)	36(56.3)	60(60.0)	
**Indication for surgery**				
Benign	34(94.4)	55(85.9)	89(89.0)	0.401
Malignant	1(2.8)	6(9.4)	7(7.0)	
Trauma	1(2.8)	3(4.7)	4(4.0)	
**ASA class**				
I	17(47.2)	30(46.9)	47(47.0)	0.600
II	14(38.9)	24(37.5)	38(38.0)	
III	5(13.9)	7(10.9)	12(12.0)	
IV	0(0.0)	3(4.7)	3(3.0)	
**Operative approach**				
Open midline	18(50.0)	33(51.6)	51(51.0)	0.881
Open non-midline	18(50.0)	31(48.8)	49(49.0)	
**Intraoperative contamination**				
Clean-contaminated	17(47.2)	38(59.4)	55(55.0)	
Contaminated	14(38.9)	19(29.7)	33(33.0)	0.502
Dirty	5(13.9)	7(10.9)	12(12.0)	
**Specialty**				
General surgery	27(75.0)	27(42.2)	54(54.0)	<0.001*
General surgery and Gynecology	0(0.0)	3(4.3)	3(3.0)	
Gynecology	0(0.0)	28(43.8)	28(28.0)	
Urology	9(25.0)	6(9.4)	15(15.0)	
**Surgical site**				
Stomach	1(2.8)	0(0.0)	1(1.0)	0.073
Small bowel	18(50.0)	21(32.8)	39(39.0)	
Large bowel	5(13.9)	4(6.3)	4(6.3)	
Pelvic collection	2(5.6)	5(7.8)	7(7.0)	
Others	10(27.8)	34(53.1)	44(44.0)	
**Hair removal at site of wound**				
Before theatre arrival	15(41.7)	51(79.7)	66(66.0)	<0.001*
In the theatre	8(22.2)	2(3.1)	10(10.0)	
Not applicable	13(36.1)	11(17.2)	24(24.0)	

The diagnosis of surgical site infection (SSI) was made in 20(20%)

**Figure 1:**
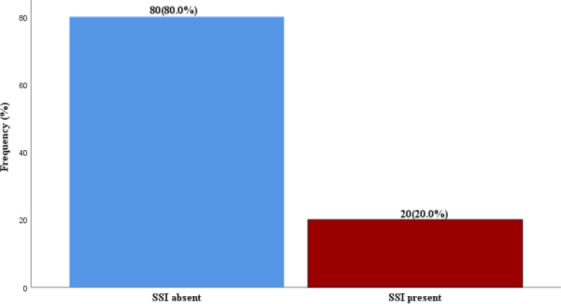
Prevalence of Surgical Site Infection (SSI)

**Figure 2:**
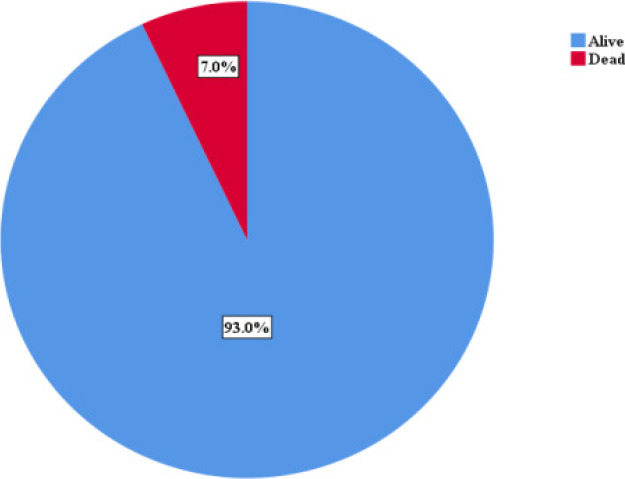
Mortality rate

All the patients were followed up for a period of 30 days; mortality outcome within the 30 day period was 7%. Figure 2

Mean age of participants with SSI was higher compared to those without SSI and they were more commonly females, 11 (11%) and the odd of having SSI also increases with age. None of the 3 (3%) patients with diabetes had SSI. Higher ASA grade (grade 111 and 1V) was associated with higher risk of SSI; 33.3% of patients with ASA 111 and 1V had SSI compared to 17.6% of patients with ASA 1 and 11. The odd of having SSI increases among patients with higher ASA grade. Most of the patients who had SSI (16 Out of the 20 patients) had emergency surgery p = 0.041. On multivariate regression, undergoing emergency surgery increases the odd of having SSI by 2 compared to having elective surgery. Surgical procedures that require open-midline approach, ranging from small bowel to colorectal surgery expectedly had higher incidence of SSI (p value 0.004). Surgical procedures requiring non- midline approach reduces the odds of having SSI more than 3 times compared to those that requires midline approach. The likelihood of SSI increases with the degree of intraoperative contamination (p <0.001). The odd of having SSI is 3 and 10 times higher in patients with contaminated and dirty wounds respectively compared with patients with clean contaminated wound (p = 0.004). In comparison to other specialties involved in the study, general surgery cases (bowel surgery) expectedly had higher incidence of SSI (p= 0.033). Surgical procedures involving gynaecology and urology specialty reduces odd of SSI more than 1 to 2 times compared to general surgery procedures. Hair removal at wound site in theatre was also associated with lesser risk of SSI compared to hair removal before theatre arrival (p = 0.035). Hair removal in theatre reduces the odds of SSI on multivariate regression (p=0.047). Table 2

**Table 2:** Relationship between SSI occurrence and other clinical parameters

	Bivariate			Multivariate	

	SSI present (n=20)	SSI absent (n=80)	p-value	Odd ratio (95% CI)	p-value
**Age in years** (Mean±SD)	40.30±14.7	35.44±15.2	0.200	1.027 (0.982-1.074)	0.250
**Gender**					
Male	9(25.0)	27(75.0)	0.349		
Female	11(17.2)	53(82.8)			
**Diabetes Mellitus status**					
Present	0(0.0)	3(100.0)	0.379		
Absent	26(20.6)	77(79.4)			
**Type of surgery**			0.041*		
Elective	4(10.0)	36(90.0)		1	
Emergency	16(26.7)	44(73.3)		2.423 (0.613-9.589)	0.207
**Indication for surgery**			0.901		
Benign	18(20.2)	71(79.8)			
Malignant	1(14.3)	6(85.7)			
Trauma	1(25.0)	3(75.0)			
**ASA class**			0.161		
I-II	15(17.6)	70(82.4)		1	
III-IV	5(33.3)	10(66.7)		2.333(0.696-7.823)	0.170
**Operative approach**			0.004*		
Open midline	16(31.4)	35(68.6)		1	
Open non-midline	4(8.2)	45(91.8)		0.318(0.088-1.145)	0.080
**Intraoperative contamination**	4(7.3)	51(92.7)	<0.001*	1	
Clean-contaminated	9(27.3)	24(41.7)		3.235(0.831-12.597)	0.091
Contaminated	7(58.3)	5(41.7)		10.320(2.062-51.650)	0.004*
Dirty					
**Specialty**					
General surgery	16(29.6)	38(70.4)	0.033*	1	
General surgery and	1(33.3)	2(66.7)		1.188(0.100-14.046)	0.892
Gynecology	3(10.7)	25(89.3)		0.285(0.075-1.080)	0.065
Gynecology	0(0.0)	15(100.0)		0.132(0.098-5.901)	0.055
Urology					
**Hair removal at site of wound**	18(27.3)	48(72.7)	0.035*		
Before theatre arrival	0(0.0)	10(100.0)		0.192(0.013-0.901)	0.047*
In the theatre	2(8.3)	22(91.7)		0.242(0.052-1.137)	0.072
Not applicable					

Out of the 7 mortality cases, 6 had emergency surgery, 5 were general surgery cases. Emergency surgery and surgical site infection increases the odds of mortality Table 3

**Table 3:** Relationship between mortality outcomes and clinical parameters

	Bivariate		Multivariate		

	Dead (n=7)	Alive (n=93)	p-value	Odd ratio (95% CI)	p-value
**Age in years** (Mean±SD)	43.14±16.8	35.90±14.8	0.224		
**Gender**					
Male	2(5.6)	34(94.4)	0.671		
Female	5(7.8)	59(92.2)			
**Diabetes Mellitus status**					
Present	0(0.0)	3(100.0)	0.629		
Absent	7(7.2)	90(92.8)			
**Type of surgery**					
Elective	1(2.5)	39(97.5)	0.150	1	
Emergency	6(10.0)	54(90.0)		3.577(0.399)	0.255
**Indication for surgery**					
Benign	5(5.6)	84(94.4)	0.244		
Malignant	1(14.3)	6(85.7)			
Trauma	1(25.0)	3(75.0)			
**ASA class**					
I-II	6(7.1)	79(92.9)	0.956		
III-IV	1(6.7)	14(93.3)			
**Operative approach**					
Open midline	4(7.8)	47(92.2)	0.736		
Open non-midline	3(6.1)	46(93.9)			
**Intraoperative contamination**					
Clean-contaminated	3(5.5)	52(94.5)	0.374		
Contaminated	2(6.1)	31(93.9)			
Dirty	2(16.7)	10(83.3)			
**SSI**					
Absent	4(5.0)	76(95.0)	0.117	1	
Present	3(15.0)	17(85.0)		2.659(0.526-13.449)	0.237
**Specialty**	0(0.0)	15(100.0)	0.619		
Urology	5(9.3)	49(90.7)			
General surgery	0(0.0)	3(100.0)			
General surgery and	2(7.1)	26(92.9)			
Gynecology					
Gynecology					
**Hair removal at site of wound**	6(9.1)	60(90.9)	0.474		
Before theatre arrival	0(0.0)	10(100.0)			
In the theatre	1(4.2)	23(95.8)			
Not applicable					

## Discussion

In this hospital-based prospective study, we sought to find out the prevalence, identifiable risk factors and outcome of surgical site infections amongst our patient who had open abdominal surgery in a tertiary hospital setting. From this study, we observed that the prevalence rate of SSI was 20.0%. This is similar to findings by Nwankwo et al (10), they reported a prevalence rate of 20.3%.

Other studies from low and middle income countries (LMIC) reported lower incidence of SSI, 17.4% and, 12.2% in Iran and Cameroun respectively^11^,^12^. The lower prevalence from these studies may be associated with the retrospective nature of their study which possibly precluded them from following up their patients for 30 days to allow for active screening for SSI. The worldwide prevalence reported by Bhangu et al was 12.3%^13^.

The prevalence rate of SSI was higher in females than males (11% vs 9%), though not statistically significant. Most studies reported higher incidence of SSI in males 5,12,14. The reason for this is not known, it has been postulated that there are sex differences in skin colonization which may be associated with differences in skin thickness, sebum production and skin pH^15^,^16^. A similar study in Nigeria reported higher prevalence in females (53.3% in females vs 46.7% in males^10^. From our study, the mean age of participants with SSI was higher than those without SSI. In addition, the odd of having SSI increases with age, though not statistically significant. Khan et al (14) also reported that the age group most affected by SSI was 41-70 years. The elderlies may have some form of compromise in their immune system; this may explains why they are at higher risk of SSI Diabetes was not associated with increased incidence of SSI in our study; this can be explained by the extremely low frequency of diabetes amongst the study participants (3%). Alkaali et al^5^ reported a higher rate of SSI among their patients with diabetes, though it was not statistically significant. However, based on the CDC guidelines, Diabetes Mellitus is considered to be one of the factors that increase the incidence of SSI^1^

There was an increase in SSI rates with increasing ASA grade, with the odd of having SSI increasing among patients with ASA grade 111 and 1V. The Centre for Disease Control and Prevention and National Nosocomial Infections Surveillance System SSI risk index recognized ASA grade as one of the three major risk factors that may contribute to the development of SSI^17^ From our study, undergoing emergency surgery was significantly associated with higher risk of SSI. Similar observation has been reported in the literature 5,18,19. Notably, general surgery (bowel surgery) cases which were also mostly performed as emergency procedures were associated with higher risk of SSI The possible explanations for this include; limited time to identify and correct potentially modifiable risk factors in the patient prior to surgical intervention^12^. Another possibility is the spillage of intestinal content into the peritoneal cavity during resection-anastomosis and the likelihood of compromise in aseptic technique in a bid to save the patient's life. The prevalence rate of SSI in our study population was found to be increasing with the wound class, with prevalence rate of up to 58.3% observed in dirty wound. Patients with contaminated and dirty wounds were 3 and 10 times more likely to develop SSI compared to contaminated wounds (p <0.001). Studies have identified dirty wound to be independently associated with SSI^3^,^20^,^21^ Outcome in this study was assessed in terms of mortality within the 30 days of follow up. We observed 7% mortality within the period. This study is limited by its small sample size which was a function of the duration of the study. In addition, taken into account the cadre of the operating surgeon, assistant, variation in techniques of different surgeons and the perioperative nurse may also be necessary to know how it affects the incidence of SSI. In our study, the general surgery and gynaecology patients had different team lead of surgeons. Surgical site infections following abdominal surgery leads to significant morbidity and mortality. Amongst many others, emergency procedures, bowel surgery, hair removal before theatre arrival and degree of intraoperative contamination are important risk factors for SSI.
